# Effects of the Methionine Hydroxyl Analogue Chelate Zinc on Antioxidant Capacity and Liver Metabolism Using ^1^H-NMR-Based Metabolomics in Aged Laying Hens

**DOI:** 10.3390/ani9110898

**Published:** 2019-11-01

**Authors:** Xi Qi, Shuxue Ma, Xing Liu, Yamin Wang, Yinglu Liu, Yupeng Gao, Yuna Min

**Affiliations:** College of Animal Science and Technology, Northwest A&F University, Xianyang 712100, Shaanxi, China; qixicc@nwsuaf.edu.cn (X.Q.); 18649362072@163.com (S.M.); 18829355190@139.com (X.L.); nwafuyummy@163.com (Y.W.); liuyinglu1996@163.com (Y.L.); gaoyupeng112@sina.com (Y.G.)

**Keywords:** MHA-Zn, aged laying hen, antioxidant capacity, metabolomics, energy metabolism

## Abstract

**Simple Summary:**

Zinc, an essential trace element for laying hens, plays an important role in biological processes, such as growth, tissue growth and repairment, skeletal development, and immune competence, which also has better effects on growth performance, biochemical indexes, and antioxidant capacity. Our previous work has shown that methionine hydroxyl analogue chelated zinc (MHA-Zn) has better effects on eggshell quality, the apparent retention of minerals and nutrients, trace element deposit, and *metallothionein (MT)* mRNA expression. The objective of the current study was to investigate the effects of different levels of MHA-Zn on antioxidant capacity and liver metabolism of aged laying hens. The results suggest that dietary supplementation of MHA-Zn levels at 80 mg/kg has better effects on antioxidant capacity and liver metabolism, as well as homeostasis of the body.

**Abstract:**

This study aimed to investigate the effects of different levels of methionine hydroxyl analogue chelated zinc (MHA-Zn) on antioxidant capacity and liver metabolism of aged laying hens. A total of 960 57-week-old layers were fed a basal diet (Zn: 35.08 mg/kg) without extra zinc for two weeks, and then allocated to four treatments consisting of eight replicates of 30 birds each for 14 weeks. Four levels of Zn (zinc sulfate (ZnSO_4_): 80 mg/kg; MHA-Zn: 20, 40, 80 mg/kg) were added to the diet. The results indicated that compared with inorganic zinc, organic zinc of 80 mg/kg has a significant advantage in improving the antioxidant capacity of aged hens, which increased the level of Cu/Zn-superoxide dismutase (SOD) and the total antioxidant capacity (T-AOC) in the serum and liver, and reduced the concentration of malondialdehyde (MDA) of laying hens. The serum albumen composition was significantly modified, meanwhile, the level of total protein, globulin, and urea increased remarkably, whereas serum glutamic-oxaloacetic transaminase decreased notably in 80 mg/kg MHA-Zn groups. Compared with the 20 mg/kg MHA-Zn group, the metabolic profile of 40 and 80 mg/kg MHA-Zn groups was higher than that of the inorganic zinc group. Furthermore, integrated key metabolic pathway analysis showed that 40 and 80 mg/kg MHA-Zn groups participated in the regulation of glutathione metabolism, glycine, serine, and threonine metabolism. Therefore, this study suggests that 40 and 80 mg/kg supplementation of MHA-Zn can increase the activity of Cu/Zn-SOD and T-AOC and decrease MDA; additionally the 80 mg/kg MHA-Zn group has better antioxidant capacity. Meanwhile, the enhanced MHA-Zn promoted methionine (Met) synthesis and protein metabolism.

## 1. Introduction

For hens, laying rate and egg quality decline during the later laying period, and these declines cause substantial economic losses. In general, after 70 weeks of age hens have retained long-term vigorous lipid metabolism, which is related to oxidant stress and decreased immunity [1]. Anti-inflammatory and antioxidant properties of zinc have long been documented [2]. Zinc is a cofactor of the antioxidant enzyme Cu, Zn-superoxide dismutase (SOD1), and Zn supplementation significantly increased glutathione peroxidase (GPX) activity through modulation of Se status [3]. However, there are many disadvantages of the inorganic form of Zn, such as high excretion, low bioavailability, high oxidation, and destruction of nutrients [4]. Recently, organic Zn products have become alternative sources. Some researchers believe that organic trace minerals can be more readily absorbed by the body, and organically bound Zn forms such as Zn-methionine (Met) and Zn-yeast [5]. Different studies have shown that dietary organic zinc reduced oxidative stress and enhanced immune response without affecting bird performance [6,7]. When broilers suffered coccidiosis vaccination, organic trace mineral complexes supplied in diets improved the immune response and lowered lesion scores [8]. Our team’s previous study also showed that 40 and 80 mg/kg methionine hydroxyl analogue chelated zinc (MHA-Zn) supplement increased eggshell quality and promoted trace element deposit [9,10]. Therefore, significant differences exist between inorganic zinc and organic zinc in the body’s antioxidant capacity, but further study is needed to clarify the different mechanisms.

Zinc deficiency significantly augmented circulating leptin concentrations [11]. Zn is known to stimulate glycolysis and inhibit gluconeogenesis, an effect that is not overcome by the presence of glucagon [12]. It is evident from previous studies that zinc plays an important role in antioxidant, lipid, and glucose homeostasis, three factors which also interact with each other. However, the differences in the metabolism of different kinds of dietary zinc are unclear. In chickens, the liver is one of the main sites of Zn bioaccumulation and plays a central role in energy metabolism. The liver is also the main organ of biotransformation and detoxification. Hence, analysis of liver metabolism activities can help clarify the mechanism of zinc metabolism in chickens. NMR data are highly reproducible and quantitative over a wide dynamic range, and they can be used to track metabolic pathways and fluxes by isotope labelling in vivo [13]. The objective of this study was to evaluate the effects of different sources and levels of zinc on antioxidant capacity and liver metabolism of aged laying hens. 

## 2. Materials and Methods

All experimental procedures were conducted in accordance with the institutional animal care and committee (IACUC) guidelines and were approved by the Animal Care and Use Committee of Northwest A&F University, Yangling, China (NWAFU-314020038). 

### 2.1. Animal Management

Hy-Line grey laying hens (*n* = 960), 57 weeks of age, were divided to four dietary treatments, each of which included eight replicates of 30 hens and 2 hens in a cage (45 cm × 45 cm × 45 cm). All laying hens were raised in an enclosed, ventilated, and conventional house with 16 h lighting and 50–70% relative humidity on average, and the house temperature was kept at 18–29 °C. The birds were fed three times per day (08:00, 11:00, 16:00), and eggs were collected at 17:00. Feed and water were offered ad libitum. The environmental conditions were the same for all groups.

### 2.2. Diet Treatment

During the first two weeks, all the groups were fed a basal diet without extra Zn to exhaust Zn accumulated in the early phase. During the following 14 weeks, four levels of Zn (inorganic Zn: 80 mg/kg; MHA-Zn: 20, 40, 80 mg/kg) were supplemented to the basal diet. The basal diet was formulated to provide all the other nutrients except Zn in accordance with nutrient recommendations from the NRC (Nutrient Requirements of Poultry, 1994) and the layer nutrition demand (China layer feeding standard: NY/T33—2004), combined with the recommendation of Hy-Line International. 

The source of inorganic zinc is pure monohydrate zinc sulfate (ZnSO_4_·H_2_O, batch number: 20150123), containing 34.5% zinc, which purchased from Hebei Yuanyuan Animal Pharmaceutical co., LTD, Shijiazhuang, China. The organic zinc is hydroxyl methionine chelated zinc (MHA-Zn, methionine mass fraction of 80%, zinc content of 12.1%, chelation rate of 90%), which was provided by Novus Intl. (Shanghai, China) and added as powder. The dietary composition and nutrient levels were listed in Table 1. The Zn concentration of the diet treatments are shown in Table 2.

### 2.3. Antioxidant and Serum Biochemical Indices

The antioxidant indices of Cu/ Zn-SOD, T-AOC, and MDA in liver and serum were measured by corresponding kits, SOD assay kits (No#A001-2) and T-AOC (No#A015-1) and MDA assay kits (No#A003-1), which were all purchased from Nanjing Jiacheng Biological Engineering Institute (Nanjing, China). The absorbance of T-AOC was measured by UV–Vis spectrophotometer (SP-752, Shanghai spectral instrument co., LTD. Shanghai, China), and the indices of Cu/ Zn-SOD and MDA in liver and serum were measured by Multiskan Spectrum (Bio-Tek, Winusky, vermont, USA). Portions of serum were sent to the hospital of Yangling demonstration area for measuring serum biochemical indices. Blood samples were centrifuged at 2500× *g* for 10 min, and the serum was separated for serum biochemical analysis using a semi-automatic clinical chemistry analyzer (CHEM-530, iCubio, Shenzhen, China) and kits were purchased from Shanghai Kehua biotechnology Co., LTD, Shanghai, China. The serum biochemical parameters included serum glutamic pyruvic transaminase (GPT), serum glutamic oxalacetic transaminase (SGOT), total protein, albumin, globulin, urea nitrogen, total cholesterol, total triglyceride, and uric acid.

### 2.4. Sample Preparation for ^1^H-NMR

Beckonert’s study on ^1^H-NMR were used for reference [14]. Prior to the measurements, freeze-dried liver samples (*n* = 32) were weighed and suspended in 1000 μL of purified water. The four seconds on/three seconds off cycling program was used (8 cycles) due to its in-solution ultrasonic extraction process (Sonics VX-130, Sonics & Materials, Inc, Newtown, CT, USA). The samples were centrifuged at 13,000 rpm for 15 min, and the aqueous layer was transferred to a 0.5-mL 3-kDa ultrafiltration filter (Merck Millipore, Inc, Darmstadt, Germany). The filtrates were collected by centrifuging the samples at 13,000 rpm for 45 min. Next, 450 μL of each filtrate was transferred to a clean 2-mL centrifuge tube, and 50 μL of 2, 2-dimethyl-2-silapentane-5-sulfonate (DSS) standard solution (Anachro Technologies, Inc, Toronto Canada) was added. The samples were mixed well before transfer to 5-mm NMR tubes (Norell, Inc, Morganton, NC, USA). The spectra were collected using an Agilent DD2 600 MHz spectrometer equipped with a triple-resonance cryoprobe. The first increment of a 2D-^1^H, ^1^H-NOESY pulse sequence was utilized for the acquisition of ^1^H-NMR data and to suppress the solvent signal. The experiments used a 100 ms mixing time along with a 990 ms pre-saturation (~80 Hz gammaB1). The spectra were collected at 25 °C, with a total of 64 scans over a period of 7 min.

### 2.5. Statistical Analysis

Results were presented as mean ± standard deviation. The main effects included the content of Cu/Zn-SOD, MDA, and T-AOC enzyme, serum biochemical indices, and liver metabolites. Data was analyzed by one-way ANOVA. Means were compared by the Duncan multiple range test (*p* < 0.05) using SPSS 21.0 software (SPSS Inc., Chicago, IL, USA).

^1^H-NMR spectroscopic profiles were obtained from the liver samples. The data were carefully phased and baseline corrected by an experienced technician using the Chenomx Processor. Multivariate statistical analysis of the data included unsupervised principal component analysis (PCA) and partial least-squares discriminant analysis (PLS-DA). Subsequently, multivariable analysis, PCA, and PLS-DA were performed using the PCA Methods Bioconductor package [15] and PLS package [16], respectively. Additionally, *t*-tests were used to analyze metabolite differences. The significantly affected metabolites between the treatment and control groups were identified by *p*-values < 0.05 obtained from *t*-tests and Variable Importance in Projection (VIP) scores > 1.

To identify the metabolic pathways of different zinc sources, we performed nuclear magnetic resonance (NMR) based metabolomics profiling of 40 an 80 mg/kg of MHA-Zn groups (C and D groups) and 80 mg/kg of ZnSO_4_ control group (A group). A pathway analysis was performed by placing the two groups of data into the pathways of the model organism to look for metabolic pathways with significant effects, and we used *t*-tests for estimation.

## 3. Results

### 3.1. Antioxidant Indices

Antioxidant indices in serum and liver were shown in Table 3. The activity of Cu/Zn-SOD and T-AOC held an increased trend while that of MDA decreased with the raise of zinc addition levels in the liver and serum. Compared with the control group, dietary supplementation with 80 mg/kg of MHA-Zn promoted the above indices in the liver and serum and significantly improved the activities of Cu/Zn-SOD (in both liver and serum) and T-AOC in the liver (*p* < 0.05). 

### 3.2. Serum Biochemical Indices

As shown in Table 4, serum level of SGOT in the 20 mg/kg MHA-Zn group increased significantly, while the level of SGOT in the 80 mg/kg MHA-Zn group decreased significantly (*p* < 0.05). The level of GPT also showed this trend but was not significant (*p* > 0.05). Total protein levels were significantly increased in the 40 and 80 mg/ kg MHA-Zn groups (*p* < 0.05), serum globulin levels were significantly increased in the 40 mg/kg MHA-Zn group (*p* < 0.05), and urea nitrogen levels were significantly increased in the 80 mg/kg MHA-Zn group (*p* < 0.05). The decreased trend of total triglyceride showed in the 80 mg/kg MHA-Zn group (*p* > 0.05).

### 3.3. Metabolite Identification

**Figure 1:**
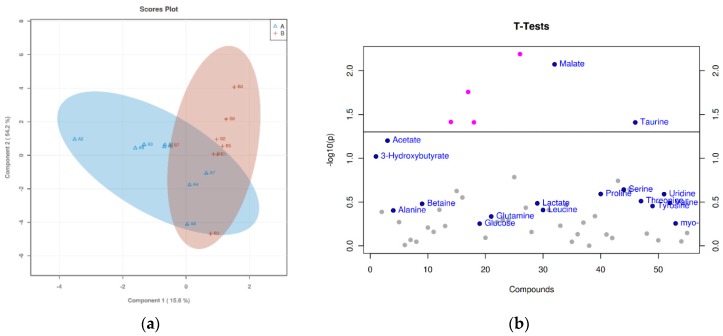
(**a**) The score plot and (**b**) *t*-test diagram of 80 mg/kg ZnSO_4_ and 20 mg/kg MHA-Zn group from the partial least-squares discriminant analysis (PLS-DA). In the above PLS-DA score chart, different groups are represented in different colors. The degree of dispersion reflects the reproducibility of the samples and the similarity of the metabolic profiles among the sample groups. The comparison group tended to separate from each other, but the trend was not significant. In the *t*-test plot, the dotted line represents *p* = 0.05, the metabolites of *p* < 0.05 are marked with red points, and the metabolites of VIP score > 1 are marked with blue points.

**Figure 2:**
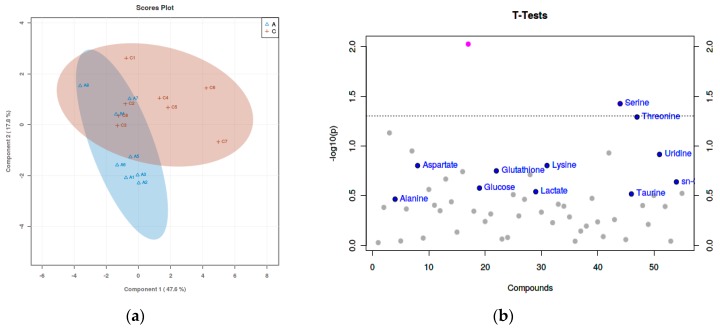
(**a**) The score plot and (**b**) *t*-test diagram of 80 mg/kg ZnSO_4_ and 40 mg/kg MHA-Zn group from the PLS-DA analysis. The result is the picture above. The comparison groups tended to separate from each other.

**Figure 3:**
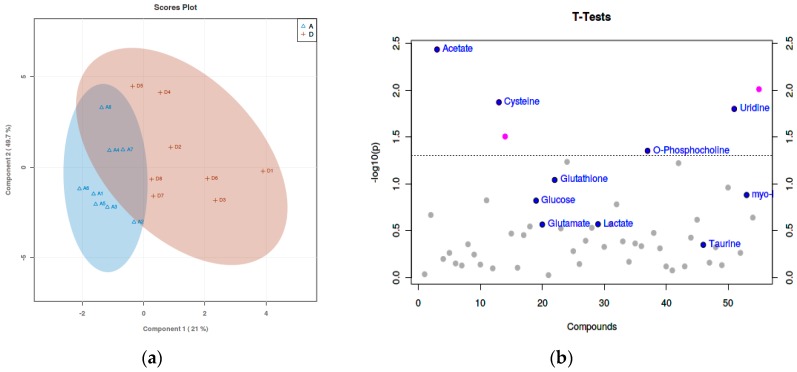
(**a**) The score plot and (**b**) *t*-test plot of 80 mg/kg ZnSO_4_ and 80 mg/kg MHA-Zn group from the PLS-DA analysis. In the above PLS-DA score chart, the comparison groups tended to separate from each other, but the trend was not significant.

Based on ^1^H-NMR analysis, the metabolites were identified. Using PLS-DA analysis to find the differential metabolites, we obtained the corresponding scores plots and *t*-test plots, as shown in Figure 1, Figure 2 and Figure 3. In the score plots, the degree of dispersion reflects the reproducibility of the samples and the similarity of the metabolic profiles among the sample groups. The results showed that the compared groups tended to be separated from each other, respectively. In the *t*-test diagram, differential metabolites with t-test analysis and VIP values greater than 1 were selected (significant difference among the variables with *p*-value < 0.05 and VIP > 1): malate, taurine, serine, threonine, uridine, acetate, o-phosphocholine, and cysteine.

### 3.4. Result of Pathway Analysis

Combining with previous studies and antioxidant indices results, the 40 and 80 mg/kg MHA-Zn groups had better antioxidant capacity. Thus we analyzed the pathway enrichment of significantly altered metabolites by referring to all metabolic pathways defined by Kyoto Encyclopedia of Genes and Genomes (KEGG). All the metabolic pathways that can produce the previously mentioned eight metabolites were screened, resulting in a total of 10 pathways (list in Table 5). Compared with the inorganic zinc group, the most significant pathway of the 40 mg/kg MHA-Zn group was glycine, serine, and threonine metabolism, and of the 80 mg/kg MHA-Zn group was glutathione metabolism; the content of metabolites of these two pathways are shown in Table 6. In the glycine, serine, and threonine metabolism pathway of 40 mg/kg MHA-Zn groups, the following compounds were detectable: pyruvic acid, choline, L-cysteine, L-serine, L-threonine, betaine, glycine, and creatine; in the glutathione metabolism pathway, L-glutamine, L-glutamic acid, and glutathione were detectable. Meanwhile, compared with the control group, the 40 mg/kg MHA-Zn group showed a significant increase trend in serine and threonine while cysteine decreased (*p* < 0.05). As the level of MHA-Zn increased, the metabolites of pyruvic acid and choline decreased, while those of methionine and betaine increased. In addition, glutamic acid, glutamine, and glutathione showed no difference between these groups (*p* > 0.05).

## 4. Discussion

Oxidative stress occurs when endogenous oxygen radicals are generated in cells, and the body counteracts or detoxifies their harmful effects through neutralization by antioxidants [17,18]. Zinc is not an antioxidant, but it is an important player in the bodies’ redox metabolism as a signal [19], and decreased zinc in cells can induce oxidative stress [20]. SOD is an enzyme whose sole function seems to be the removal of superoxide anions [21]. Zinc is necessary for the structure and function of Cu/Zn-SOD, which comprises 90% of total SOD and protects tissues from oxidative damage [22,23]. Dietary zinc supplementation can increase the expression of Cu/Zn-SOD in the liver and serum of broilers. Several studies have shown that elevated expression of Cu/Zn-SOD alone or in combination with catalase likewise results in enhanced stress tolerance and protection against acute oxidative stress [24]. MDA is an indicator of lipid peroxidation that indicates the oxidative damage of cells [25]. Regardless of the source, supplementation of Zn can promote the formation of metallothionein, remove free radicals in the body, inhibit lipid peroxidation in vivo, reduce the content of MDA, and increase Cu/Zn-SOD [26]. In our previous studies, we found that 40 and 80 mg/kg MHA-Zn group had a higher content of zinc in the liver than the control group, and the level of *metallothionein* (MT) mRNA expression in the liver was highest in the 80 mg/kg MHA-Zn group [10]. This means MHA-Zn has higher observation and nutrition. This present study found that, compared with inorganic zinc, increased Cu/Zn-SOD and T-AOC in liver and serum and decreased MDA and the ND, obtained with 80 mg/kg of MHA-Zn, provided the older laying hens with improved antioxidant capacity.

Serum biochemical index is sensitive to reflect the physiological metabolism of the body state, which is a concentrated reflection of the situation of the nutrient metabolism in the body. Serum total protein is affected by various factors such as protein synthesis rate, protein loss, intake, and metabolic rate. Zinc plays an important role in the enzyme system and is involved in protein synthesis, carbohydrate metabolism, and many other biochemical reactions. Most globulins have immune function and immunoglobulin has antibody activity in human serum [27]. In this research, organic zinc supplement significantly improved total protein and globulin. Previous studies also proved that dietary Zn supplementation significantly affected serum total protein, albumin, glucose, immunoglobulin, and the performance of laying hens [9,28,29]. Urea nitrogen is a product of protein degradation in the body. The increase of nitrogen content indicates the enhancement of protein catabolism. This study found that with 80 mg/kg MHA-Zn supplement, serum urea nitrogen had a significant increase trend. In addition, some studies found that compared with no supplement zinc group, organic zinc group decreased the level of serum triglyceride and low density lipoprotein (LDL) cholesterol [29,30]. However, we found that Zn had no difference in promoting lipid metabolism.

In this study, the ^1^H-NMR-based metabolomics approach revealed metabolic changes and identified significant metabolic pathways that reflect different sources of zinc; two of these pathways, glycine, serine and threonine metabolism and glutathione metabolism, showed a significant connection with Zn signaling. These two pathways and metabolite markers are related to oxidative stress, energy, and amino acid metabolism. In the glycine, serine, and threonine pathways, threonine in the liver of 40 mg/kg MHA-Zn group had higher levels than the control group. In many studies, dietary threonine has shown antioxidant functions, and can improve growth production, serum biochemical indexes, and antioxidant capacities in poultry [31,32]. An adequate dietary threonine level also significantly improved immune responses and regulated gene expression of antioxidant–immune–cytokine-related signaling molecules in juvenile blunt snout bream [33]. A similar study found that threonine significantly increased Cu/Zn-SOD and mRNA levels of Cu/Zn-SOD in poultry [34].

Glycine, as a necessary amino acid in poultry, participates in the formation of uric acid [35]. Gly can be metabolized from choline if L-homocysteine is available [36]; Ser has been found to be equally effective in meeting the functions of Gly when considered on an equimolar basis [37], and the metabolism of glycine and serine produces carbon units that serve as materials for purine and pyrimidine biosynthesis.

Methionine (Met) is an essential amino-acid (EAA) and an important methyl donor with crucial roles in 1-carbon metabolism and transsulfuration. Adding methionine (Met) may promote flux through the transsulfuration pathway, thereby the increasing production of S-adenosyl-Met (SAM) produces the antioxidant glutathione (GSH) and taurine with the consumption of ATP. Increased antioxidant production may help reduce oxidative stress and inflammation caused by the production of reactive oxygen species in the liver [38]. In addition, serine supports the Met cycle through de novo ATP synthesis [39]. Choline also supports the Met cycle through its metabolite betaine, which is used to produce Met [40]. This reaction produces N, n-dimethyl glycine, which is metabolized first to creatine and then to glycine [41]. Therefore, it means that the levels of methionine, serine, threonine, and betaine increased in the 40 mg/kg MHA-Zn group promoted Met synthesis. That may be due to the higher content of hydroxyl methionine which was above 80% of the MHA-Zn, so when we enhanced MHA-Zn, we supplied more methionine in the layer diet. However, in the 80 mg/kg MHA-Zn group, the level of glutamine, glutamic acid, and glutathione of the glutathione metabolism pathway was no different compared with the control group. In the metabolism result, the providing precursors for GSH synthesis, glutamate, and cysteine, these two metabolites had a decreasing trend. That did not match with the result of antioxidant indices, in which the activity of Cu/Zn-SOD and T-AOC increased in the organic zinc group. One reasoning maybe that compared with the 80 mg/kg ZnSO_4_ group, the 80 mg/kg MHA-Zn group had enhanced hydroxyl methionine to promote Met synthesis rather than to compound GSH by Met which process consumed a lot ATP, which is the limit factor for the Met cycle. 

Studies have shown that zinc can reduce triglycerides and affect both fat synthesis and glucose metabolism [30,42]. In a rat study, the content of triglycerides (TGs) was decreased, and high-density lipoprotein cholesterol (HDL-C) was increased by the addition of dietary zinc, verifying the promoting effect of zinc on lipid metabolism [30]. Choline is oxidized to betaine mainly in the liver and kidney [43,44]. In the late stage of egg production, fat synthesis in the liver is more vigorous and more likely to increase the risk of inflammation, and organic zinc supplementation can increase the level of lipid metabolism-related enzymes and gene expression in many layers or broilers [45,46]. Due to the decreased level of choline and increased level of betaine, MHA-Zn still plays a role in effecting lipid metabolism, but this trend is not significant.

## 5. Conclusions

The results demonstrate that 40 and 80 mg/kg supplementation of MHA-Zn can increase the activity of Cu/Zn-SOD and T-AOC and decrease MDA, and the 80 mg/kg MHA-Zn group has better antioxidant capacity. Meanwhile, the enhanced MHA-Zn promoted Met synthesis and protein metabolism.

## Figures and Tables

**Table 1:** Composition and nutrient levels of the basal diet (air-dry basis).

Ingredients	%	Nutrients	%
Corn	58.50	Metabolic energy (MJ/kg)	12.29
Soybean meal	23.00	Crude protein ^2^	15.47
Ground limestone	8.74	Calcium ^2^	3.60
CaHPO_4_	1.10	Total phosphorus	0.62
Wheat bran	7.50	Available phosphorus	0.32
Soybean oil	0.50	Lysine	0.94
NaCl	0.24	Methionine	0.44
Methionine	0.16	Threonine	0.70
Threonine	0.03	Zinc ^2^ (mg/kg)	35.08
Lysine	0.05		
Premix ^1^	0.18		
Total	100.00		

^1^ Supplied the following per kilogram of diet: Vitamin A 10,000 IU, VD3 31 800 IU, VE 10 IU, VK 10 mg, VB 125 μg, thiamine l mg, riboflavin 4.5 mg, calcium pantothenate 50 mg, niacin 24.5 mg, pyridoxine 5 mg, biotin 1 mg, folic acid 1 mg, choline 500 mg, Mn (as manganese sulfate) 60 mg, I (as calcium iodate) 0.4 mg, Fe (as ferrous sulfate) 60 mg, Cu (as copper sulfate) 8 mg, Se (sodium selenite) 0.30 mg. ^2^ Analyzed values, each value based on triplicate determinations.

**Table 2:** Concentrations of zinc in experimental treatment diets (air-dry basis).

Dietary Zinc Supplementation	Calculated (mg/kg)	Analyzed (mg/kg)
80 mg/kg ZnSO_4_	115.08	115.53
20 mg/kg MHA-Zn	55.08	55.38
40 mg/kg MHA-Zn	75.08	75.97
80 mg/kg MHA-Zn	115.08	114.18

Value based on triplicate determinations; ZnSO_4_: zinc sulphate; MHA-Zn: methionine hydroxy analog chelate zinc, methionine yielded 80%.

**Table 3:** Effects of MHA-Zn on the antioxidant parameters of the serum and liver (72 weeks) ^1^.

Items		80 mg/kg ZnSO_4_	20 mg/kg MHA-Zn	40 mg/kg MHA-Zn	80 mg/kg MHA-Zn	*p*-Value
Cu/Zn-SOD	Liver (U/mgprot)	66.62 ± 2.80 ^b^	61.25 ± 7.92 ^b^	67.90 ± 5.82 ^b^	75.23 ± 3.97 ^a^	0.003
	Serum (U/mL)	60.09 ± 1.43 ^b^	54.45 ± 3.25 ^c^	56.04 ± 1.22 ^c^	64.89 ± 2.35 ^a^	<0.001
T-AOC	Liver (U/gprot)	1.76 ± 0.18 ^b^	1.49 ± 0.24 ^b^	1.81 ± 0.17 ^ab^	2.12 ± 0.40 ^a^	0.006
	Serum (U/mL)	9.31 ± 0.28 ^ab^	7.73 ± 0.58 ^c^	8.98 ± 0.57 ^b^	9.81 ± 0.23 ^a^	<0.001
MDA	Liver (nmol/gprot)	3.41 ± 0.34 ^ab^	3.72 ± 0.37 ^a^	3.26 ± 0.35 ^ab^	3.04 ± 0.61 ^b^	0.088
	Serum (nmol/mL)	3.57 ± 0.16 ^bc^	4.36 ± 0.42 ^a^	4.03 ± 0.46 ^ab^	3.48 ± 0.49 ^c^	0.008

MHA-Zn: methionine hydroxy analogue chelate zinc. ^a, b c^ Different superscripts within the same row are significantly different (*p* < 0.05). ^1^ The data are represented as the means of eight replicates per treatment. The values are expressed as the means ± SD.

**Table 4:** Effects of MHA-Zn on serum biochemical indexes of laying hens.

Item	80 mg/kg ZnSO_4_	20 mg/kg MHA-Zn	40 mg/kg MHA-Zn	80 mg/kg MHA-Zn	*p*-Value
GPT/mmol/L	1.53 ± 0.67	2.26 ± 0.15	1.76 ± 0.66	1.43 ± 0.15	0.229
SGOT/mmol/L	187.2 ± 1.35 ^ab^	214.2 ± 13.15 ^a^	189.7 ± 13.05 ^ab^	157.7 ± 23.72 ^b^	0.018
TP (g/L)	42 ± 2.86 ^ab^	35.83 ± 5.38 ^b^	47.67 ± 3.02 ^a^	43.86 ± 4.11 ^a^	0.037
ALB (g/L)	17.43 ± 1.05	14.9 ± 2.69	16.2 ± 1.68	17.5 ± 1.18	0.302
GLB (g/L)	24.57 ± 3.85 ^b^	20.93 ± 2.69 ^b^	31.47 ± 1.79 ^a^	26.37 ± 3.25 ^ab^	0.016
ALB/GLB (%)	0.67 ± 0.11	0.71 ± 0.04	0.59 ± 0.09	0.67 ± 0.06	0.38
UN/mmol/L	0.29 ± 0.07 ^b^	0.24 ± 0.09 ^b^	0.49 ± 0.28 ^b^	1.06 ± 0.26 ^a^	0.025
TC/mmol/L	2.25 ± 0.18	1.89 ± 0.27	2.24 ± 0.5	2.31 ± 0.58	0.625
TG/mmol/L	13.93 ± 1.22	13.97 ± 3.5	13.23 ± 0.37	10.06 ± 2.18	0.163
UA/mmol/L	0.067 ± 0.011	0.101 ± 0.016	0.089 ± 0.037	0.091 ± 0.009	0.343

GPT: serum glutamic pyruvic transaminase; SGOT: serum glutamic oxalacetic transaminase; TP: total protein; ALB: albumin; GLB: globulin; UN: urea nitrogen; TC: total cholesterol; TG: total triglyceride; UA: uric acid. ^a, b^ Different superscripts within the same row are significantly different (*p* < 0.05).

**Table 5:** Selected Kyoto Encyclopedia of Genes and Genomes (KEGG) pathway.

Groups	Pathway Name	Match Status	*p*-Value	-log(p)	Holm p	FDR	Impact
A/C	Glycine, serine, and threonine metabolism	8/33	0.05481	2.9039	1	0.64662	0.5867
	Beta-alanine metabolism	4/20	0.17964	1.7168	1	0.64662	0.4510
	Alanine, aspartate, and glutamate metabolism	9/23	0.36335	1.0124	1	0.64662	0.6733
	Glutathione metabolism	5/26	0.46206	0.77205	1	0.64662	0.4183
	Phenylalanine, tyrosine, and tryptophan biosynthesis	2/4	0.48015	0.73366	1	0.64662	1
A/D	Glutathione metabolism	5/26	0.16582	1.7969	1	0.56967	0.4183
	Beta-alanine metabolism	4/20	0.20855	1.5676	1	0.56967	0.4510
	D-glutamine and D-glutamate metabolism	2/5	0.39423	0.93081	1	0.64063	1
	Glycine, serine, and threonine metabolism	8/33	0.4149	0.87971	1	0.64684	0.5867
	Alanine, aspartate, and glutamate metabolism	9/23	0.47665	0.74098	1	0.64684	0.6733

A: 80 mg/kg inorganic Zn; C and D: 40 and 80 mg/kg of MHA-Zn, respectively. Pathway analysis was carried out by combining two sets of data into the pathway of model organisms (*Gallus* (chicken)). **FDR:** The false discovery rate

**Table 6:** Effect of MHA-Zn on the concentration of differentially expressed liver metabolites in laying hens.

Item	80 mg/kg ZnSO_4_	20 mg/kg MHA-Zn	40 mg/kg MHA-Zn	80 mg/kg MHA-Zn	*p*-Value
Pyruvic acid (mmol/L)	0.066 ± 0.010	0.046 ± 0.007	0.057 ± 0.006	0.060 ± 0.014	0.237
Glycine (mmol/L)	0.601 ± 0.040	0.663 ± 0.076	0.592 ± 0.038	0.516 ± 0.065	0.114
Serine (mmol/L)	0.518 ± 0.050 ^b^	0.652 ± 0.094 ^a^	0.679 ± 0.047 ^a^	0.605 ± 0.078 ^b^	0.040
Cysteine (mmol/L)	0.096 ± 0.011 ^a^	0.086 ± 0.010 ^a^	0.077 ± 0.008 ^b^	0.056 ± 0.007 ^b^	0.042
Choline (mmol/L)	0.079 ± 0.011	0.085 ± 0.009	0.065 ± 0.009	0.057 ± 0.009	0.199
Threonine (mmol/L)	0.359 ± 0.035 ^b^	0.446 ± 0.073 ^ab^	0.483 ± 0.045 ^a^	0.386 ± 0.053 ^b^	0.045
Creatine (mmol/L)	0.057 ± 0.008	0.049 ± 0.005	0.076 ± 0.009	0.054 ± 0.008	0.148
Betaine (mmol/L)	0.229 ± 0.036	0.293 ± 0.052	0.240 ± 0.040	0.260 ± 0.039	0.342
Methionine (mmol/L)	0.143 ± 0.012	0.152 ± 0.021	0.168 ± 0.015	0.172 ± 0.012	0.368
Glutamic acid (mmol/L)	0.774 ± 0.066	0.806 ± 0.013	0.726 ± 0.055	0.655 ± 0.080	0.485
Glutamine (mmol/L)	0.376 ± 0.045	0.431 ± 0.055	0.527 ± 0.050	0.382 ± 0.047	0.163
Glutathione (mmol/L)	0.067 ± 0.010	0.057 ± 0.012	0.048 ± 0.008	0.042 ± 0.009	0.134

^a, b^ Means with different superscripts within the same row are significantly different (*p* < 0.05).
